# BBGD: an online database for blueberry genomic data

**DOI:** 10.1186/1471-2229-7-5

**Published:** 2007-01-30

**Authors:** Nadim W Alkharouf, Anik L Dhanaraj, Dhananjay Naik, Chris Overall, Benjamin F Matthews, Lisa J Rowland

**Affiliations:** 1 Department of computer and information sciences, Towson University, 7800 York Road, Towson, Maryland, 21252, USA; 2 Fruit Laboratory, USDA/ARS, Henry A. Wallace Beltsville Agricultural Research Center, Bldg. 010A, BARC-West, 10300 Baltimore Ave., Beltsville, MD 20705, USA; 3 Soybean Genomics and Improvement Laboratory, USDA/ARS, Henry A. Wallace Beltsville Agricultural Research Center, Bldg. 010A, BARC-West, 10300 Baltimore Ave., Beltsville, MD 20705, USA; 4 George Mason University, School of Computational Sciences, Manassas, VA 20110, USA

## Abstract

**Background:**

Blueberry is a member of the *Ericaceae *family, which also includes closely related cranberry and more distantly related rhododendron, azalea, and mountain laurel. Blueberry is a major berry crop in the United States, and one that has great nutritional and economical value. Extreme low temperatures, however, reduce crop yield and cause major losses to US farmers. A better understanding of the genes and biochemical pathways that are up- or down-regulated during cold acclimation is needed to produce blueberry cultivars with enhanced cold hardiness. To that end, the blueberry genomics database (BBDG) was developed. Along with the analysis tools and web-based query interfaces, the database serves both the broader *Ericaceae *research community and the blueberry research community specifically by making available ESTs and gene expression data in searchable formats and in elucidating the underlying mechanisms of cold acclimation and freeze tolerance in blueberry.

**Description:**

BBGD is the world's first database for blueberry genomics. BBGD is both a sequence and gene expression database. It stores both EST and microarray data and allows scientists to correlate expression profiles with gene function. BBGD is a public online database. Presently, the main focus of the database is the identification of genes in blueberry that are significantly induced or suppressed after low temperature exposure.

**Conclusion:**

By using the database, researchers have developed EST-based markers for mapping and have identified a number of "candidate" cold tolerance genes that are highly expressed in blueberry flower buds after exposure to low temperatures.

## Background

Blueberry (*Vaccinium corymbosum*) is one of the major berry crops grown in the United States [1]. North America, in fact, is the world's leading blueberry producer, accounting for nearly 90% of world production at the present time. Total area devoted to growing commercial blueberries in North America is approximately 74,000 hectares. Blueberry is a high value crop, often times grown in acidic and imperfectly drained soils that would otherwise be considered unfit for agricultural production [2]. Blueberry is also an important fruit crop because of its nutritional value. Of all fresh fruits and vegetables, blueberries are one of the richest sources of antioxidants [3]. Blueberry is a model organism for the heath family *Ericaceae*, which also includes the economically important, closely related cranberry as well as the economically important, more distantly related ornamentals, rhododendron, azalea, and mountain laurel. For all these related species, genomic studies, including EST generation and microarray analyses, are lacking or completely absent. Functional genomic studies on berry crops are lacking, especially studies dealing with the molecular impacts of low temperature on berry crop yield. Low temperature extremes reduce blueberry yields and impact the profitability and competitiveness of U.S. producers. Enhanced cold tolerance during the winter and early spring of elite varieties would be of great value to the blueberry industry. The Blueberry Genomics Database [4] is a public database that links blueberry expressed sequence tags (ESTs) with gene expression data and provides embedded analytical tools for data mining. BBGD was established in 2005 to serve as a sequence and microarray database for the blueberry community with its primary focus to store and analyze ESTs and microarray data generated from experiments aimed at studying cold acclimation and mid-winter hardiness of blueberry. The ultimate goal of these experiments is to apply the information to develop more cold hardy cultivars. The database allows for the correlation of expression levels and function by linking the EST data with the microarray results, since many of the ESTs were printed on the microarray slides. BBGD also serves as a means of novel gene discovery through EST analysis. Numerous applications to conduct statistical analysis on DNA microarray data have been integrated into BBGD for rapid data analysis. Analytical tools include t-tests to detect significantly induced/suppressed genes and online analytical processing (OLAP) to find correlations and relationships in data sets. These tools have been integrated into the database, thereby eliminating the need for third-party software.

## Construction and content

BBGD is a relational database built on SQLServer2000 and is housed at the Beltsville Agricultural Research Center in Beltsville, MD, USA. The database was implemented on a server running Windows 2000 Server and Internet Information Server (IIS 5.0). The web interface uses active server pages (ASP) and ASP.Net scripts, written in visual basic, to query the backend database. BBGD is divided into two separate but related entities, a sequence database and a microarray database. This allows for the correlation of gene function, deduced from the EST data, with expression levels, deduced from the microarray data. The bulk of the EST and microarray data held at the BBGD currently deals with identifying cold-responsive genes in blueberry flower buds.

## Utility and discussion

**Figure 1:**
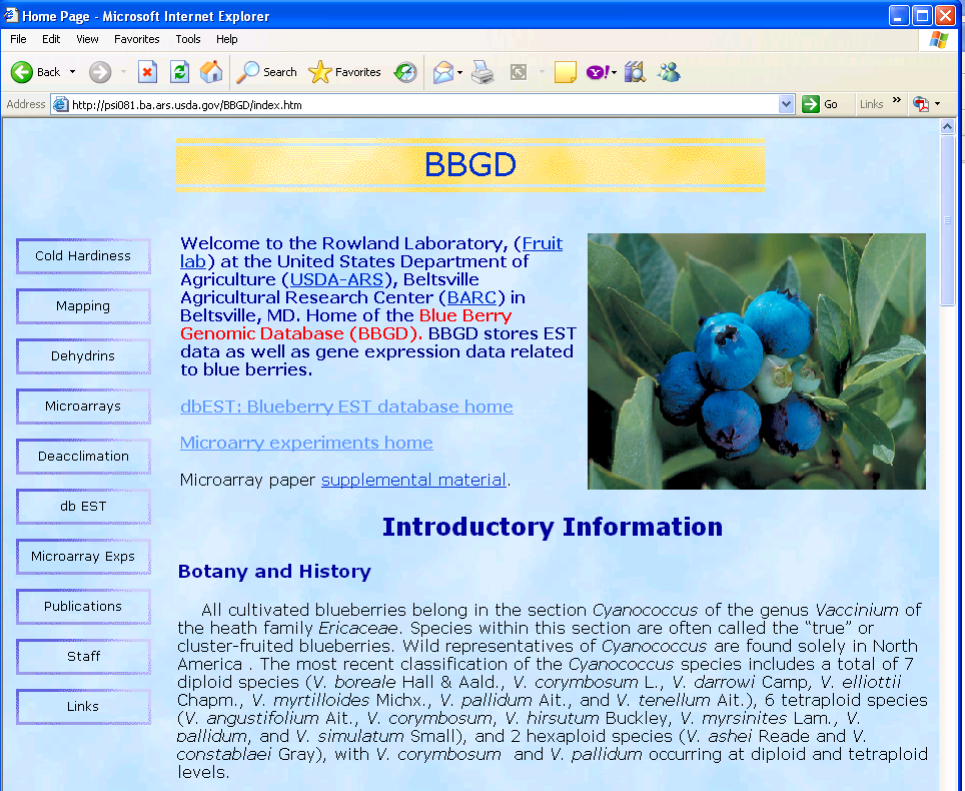
A snap shot of BBGD's main web page that shows the wealth of information that is available through the web site and the general capabilities of the database.

The BBGD web site acts as a gateway to the microarray and EST sequencing projects that have been implemented to identify cold-responsive genes in blueberry (fig. 1). In addition, it provides a wealth of general information on blueberry for the public.

### Microarray experiments

**Figure 2:**
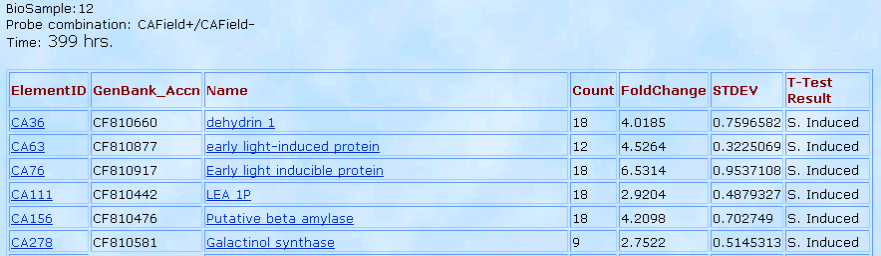
A snap shot showing the results obtained when querying BBGD for genes that had an expression ratio of >= 2 fold. Among the fields that are returned are results from standard deviation calculations (STDEV) and T-test calculations summary (significantly induced/suppressed or no change). Users can get the sequence by clicking on the clone ID and/or can query PubMed for relevant articles by clicking on the gene name.

DNA microarrays allow for the measurement of mRNA expression levels for thousands of genes at a time. The microarray portion of the database stores microarray experiments that measure gene expression changes in blueberry flower buds across a number of time points after low temperature exposure in the field and the cold room environment [5]. A list of slides printed during one of the experiments is illustrated in table 1. A list of genes that were printed on the slides are available as a supplement [see Additional file 1]. A number of web based applications have been implemented that allow users to query the microarray experiments from anywhere using a web browser and an internet connection. Users can choose to query a specific time point (fig. 2), across all or selected time points, or query by gene name, ID or GenBank accession number. Users can also conduct advanced queries to find genes that have similar expression in different experiments and/or biological samples. Results from cluster analysis and online analytical processing (OLAP) [6,7] are also displayed.

### Sequence database

**Figure 3:**
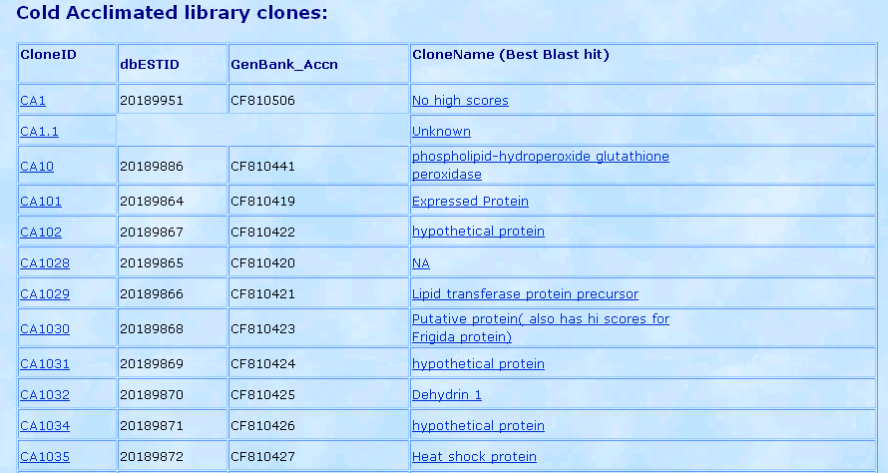
The sequence database allows users to browse a specific library in table format. This snap shot represents part of the table that was returned by the database. Users can get the sequence by clicking on the clone ID and/or can query PubMed for relevant articles by clicking on the gene name.

**Figure 4:**
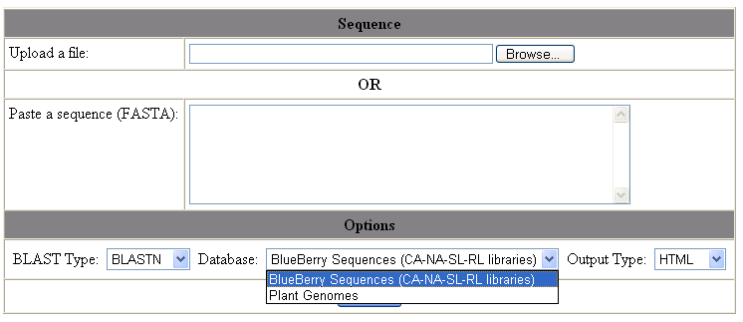
The Blast application allows users to cut and paste a sequence or to select a file containing a FASTA sequence and Blast the in-house sequences stored in BBGD or a collection of EST and genomic sequences from all plant species (kingdom Viridiplantae).

The sequence database provides access to EST sequences stored at BBGD. With the development of high-throughput DNA sequencing technologies, EST analysis has become a rapid and relatively inexpensive way to identify genes, proteins, and metabolic pathways through homology with other sequence data repositories such as GenBank. Analysis of ESTs can provide an overall picture of transcripts involved in organ or tissue development. In BBGD, all relevant information about every EST is stored; including the cloning vector and bacterial host strain, insert size, dbEST ID, GenBank accession number, and Blast results, which include E-value, score and identity percentage. Perl scripts were written to extract this information from the Blast results and are available through the authors. The database also contains results of EST analysis [8] and contig assembly for the libraries that were sequenced, along with graphical representations and charts. Table 2 depicts the libraries that were locally constructed; contig assembly and analysis results are provided on the web site. SeqMan from DNAStar Inc (Madison, WI) was used for contig assembly and clustering. Like the microarray portion, the sequence database allows users to query by gene name, ID or GenBank accession number. Users can also browse a specific library in a table format (fig. 3). The database was extremely useful in the identification and characterization of transcripts that are highly expressed during cold acclimation [8,9] and in the development of EST-based markers for mapping cold tolerance in blueberry [10]. A Blast application (fig. 4) was developed that provides users with a means to Blast a sequence of interest against the ESTs stored in BBGD and/or a collection of sequence data comprising EST and genomic sequences from all plant species (kingdom Viridiplantae). Since the blueberry genome has not been sequenced, EST sequences such as the ones stored in BBGD will play an important role in gene identification and discovery, as they have in other organisms [11-14].

## Conclusion

As a result of BBGD and the associated analysis tools, genes potentially involved in cold acclimation in blueberry were identified [5,8]. From the microarray experiments a number of genes were found to be up-regulated across all measured time points. To name a few, among them were stress/defense related genes dehydrins and GRPF1, cell structure genes, and auxin-mediated signaling pathway genes such as protein kinase PINOID [5]. From the EST analysis alone, monooxygenase, dehydrins, beta amylase, galactinol synthase, and heat shock proteins, among others, were identified as being highly expressed during cold acclimation, demonstrating how analysis of ESTs was an effective strategy to identify candidate cold acclimation-responsive transcripts in blueberry [8]. Blueberry is an important small fruit crop, and these types of studies on cold acclimation in flower buds will go a long way toward achieving our ultimate goal of producing more cold hardy cultivars.

## Future perspectives

Work is underway to add data on the current status of the blueberry genetic linkage maps and EST-PCR markers being used for mapping.

## Availability and requirements

BBGD is accessible from  Sequence and microarray data can be downloaded and the manager of the database can be contacted by email at nalkharouf@towson.edu.

## Abbreviations

EST – Expressed Sequence Tag; BBGD – Blueberry Genomics Database.

## Authors' contributions

NWK designed and developed the database, he also developed most of the web interface. CO developed the blast application, while ALD, DN, BFM and LJR contributed to the generation of the EST and microarray data. All authors have read and approved the final manuscript.

## Supplementary Material

Additional File 1 List of genes printed on microarray slides. The supplemental table lists all the genes that were printed on the microarray slides that were involved in the blue berry cold hardiness experiments [5]. Click here for file

## Figures and Tables

**Table 1:** List of slides printed during one of the microarray experiments stored in BBGD. Probe combination refers to the RNA samples that were used in hybridization, and combination order refers to the dye that was used to label each sample.

**Slide number**	**Biological sample**	**Probe Combination**	**Combination Order**	**Time point (hours)**
24	1	CAColdRm-/CAColdRm-	647/555	0
25	1	CAColdRm+/CAColdRm-	647/555	500
26	1	CAColdRm+/CAColdRm-	647/555	1000
27	1	CAColdRm-/CAColdRm-	555/647	0
28	1	CAColdRm+/CAColdRm-	555/647	500
29	1	CAColdRm+/CAColdRm-	555/647	1000
36	1	CAField-/CAField-	647/555	0
37	1	CAField+/CAField-	647/555	67
38	1	CAField+/CAField-	647/555	399
39	1	CAField+/CAField-	647/555	779
40	1	CAField+/CAField-	647/555	1234
41	1	CAField-/CAField-	555/647	0
42	1	CAField+/CAField-	555/647	67
43	1	CAField+/CAField-	555/647	399
44	1	CAField+/CAField-	555/647	779
45	1	CAField+/CAField-	555/647	1234
46	1	CAField-/CAField-_Tifblue	647/555	0
47	1	CAField+/CAField-_Tifblue	647/555	67
48	1	CAField+/CAField-_Tifblue	647/555	399
49	1	CAField+/CAField-_Tifblue	647/555	779
50	1	CAField+/CAField-_Tifblue	647/555	1234
51	1	CAField-/CAField-_Tifblue	555/647	0
52	1	CAField+/CAField-_Tifblue	555/647	67
53	1	CAField+/CAField-_Tifblue	555/647	399
54	1	CAField+/CAField-_Tifblue	555/647	779
55	1	CAField+/CAField-_Tifblue	555/647	1234
66	1	CAField+/CAField-_Tifblue	647/555	67
67	1	CAColdRm-/CAColdRm-_Tifblue	647/555	0
68	1	CAColdRm+/CAColdRm-_Tifblue	647/555	500
69	1	CAColdRm+/CAColdRm-_Tifblue	555/647	500
70	2	CAColdRm-/CAColdRm-_Tifblue	647/555	0
71	2	CAColdRm+/CAColdRm-_Tifblue	647/555	500
72	2	CAColdRm-/CAColdRm-_Tifblue	555/647	0
73	2	CAColdRm+/CAColdRm-_Tifblue	555/647	500
56	2	CAField-/CAField-_Tifblue	647/555	0
57	2	CAField+/CAField-_Tifblue	647/555	67
58	2	CAField+/CAField-_Tifblue	647/555	399
59	2	CAField+/CAField-_Tifblue	647/555	779
60	2	CAField+/CAField-_Tifblue	647/555	1234
61	2	CAField-/CAField-_Tifblue	555/647	0
62	2	CAField+/CAField-_Tifblue	555/647	67
63	2	CAField+/CAField-_Tifblue	555/647	399
64	2	CAField+/CAField-_Tifblue	555/647	779
65	2	CAField+/CAField-_Tifblue	555/647	1234
30	2	CAColdRm-/CAColdRm-	647/555	0
31	2	CAColdRm+/CAColdRm-	647/555	500
32	2	CAColdRm+/CAColdRm-	647/555	1000
33	2	CAColdRm-/CAColdRm-	555/647	0
34	2	CAColdRm+/CAColdRm-	555/647	500
35	2	CAColdRm+/CAColdRm-	555/647	1000
14	2	CAField-/CAField-	647/555	0
15	2	CAField+/CAField-	647/555	67
16	2	CAField+/CAField-	647/555	399
17	2	CAField+/CAField-	647/555	779
18	2	CAField+/CAField-	647/555	1234
19	2	CAField-/CAField-	555/647	0
20	2	CAField+/CAField-	555/647	67
21	2	CAField+/CAField-	555/647	399
22	2	CAField+/CAField-	555/647	779
23	2	CAField+/CAField-	555/647	1234

**Table 2:** List of the locally constructed libraries stored in BBGD and the number of ESTs in each.

**Library**	**Number of ESTs**
Cold acclimated	1312
Non-acclimated	1241
Forward substractive library	586
Reverse substractive library	287
